# *Phyllomedusa bicolor* skin secretion and the *Kambô* ritual

**DOI:** 10.1186/1678-9199-20-40

**Published:** 2014-09-02

**Authors:** Paul S den Brave, Eugéne Bruins, Maarten W G A Bronkhorst

**Affiliations:** 1 Department of Surgery, Bronovo Hospital, Bronovolaan 5, 2597 AX Den Haag, The Netherlands; 2 CEO Zoological Services, Ede, The Netherlands

## Abstract

The ritual of Kambô or Sapo is a type of voluntary envenomation. During this purification ritual a shaman healer, from various South American countries, deliberately burns the right shoulder with a glowing stick from a fireplace. Excretions of Phyllomedusa bicolor (or Giant Leaf Frog, Kambô or Sapo) are then applied to these fresh wounds. This ritual is used as a means of purification of the body, supposedly brings luck to hunters, increases stamina and enhances physical and sexual strength. All the peripheral and most of the central effects of the secretion can be ascribed to the exceptionally high content of active peptides, easily absorbed through burned skin. This article describes the ritual and the bio-active peptides from the secretion.

## Dear Editor of JVATiTD

*Kambô* or *Sapo* is a purification ritual performed in numerous South American countries including Bolivia, Brazil, Colombia, Peru, French Guiana, Suriname and Venezuela. During the ceremony, a shaman healer deliberately burns the right shoulder of the participant with a burning stick from a fireplace. Subsequently, secretion of *Phyllomedusa bicolor* (giant leaf frog or *kambô*) is applied to the fresh wounds. The aim of this ritual is the purification of the body. All the peripheral and most of the central effects of the secretion can be ascribed to its exceptionally high content of active peptides easily absorbed through wounded skin [1].

**Figure 1:**
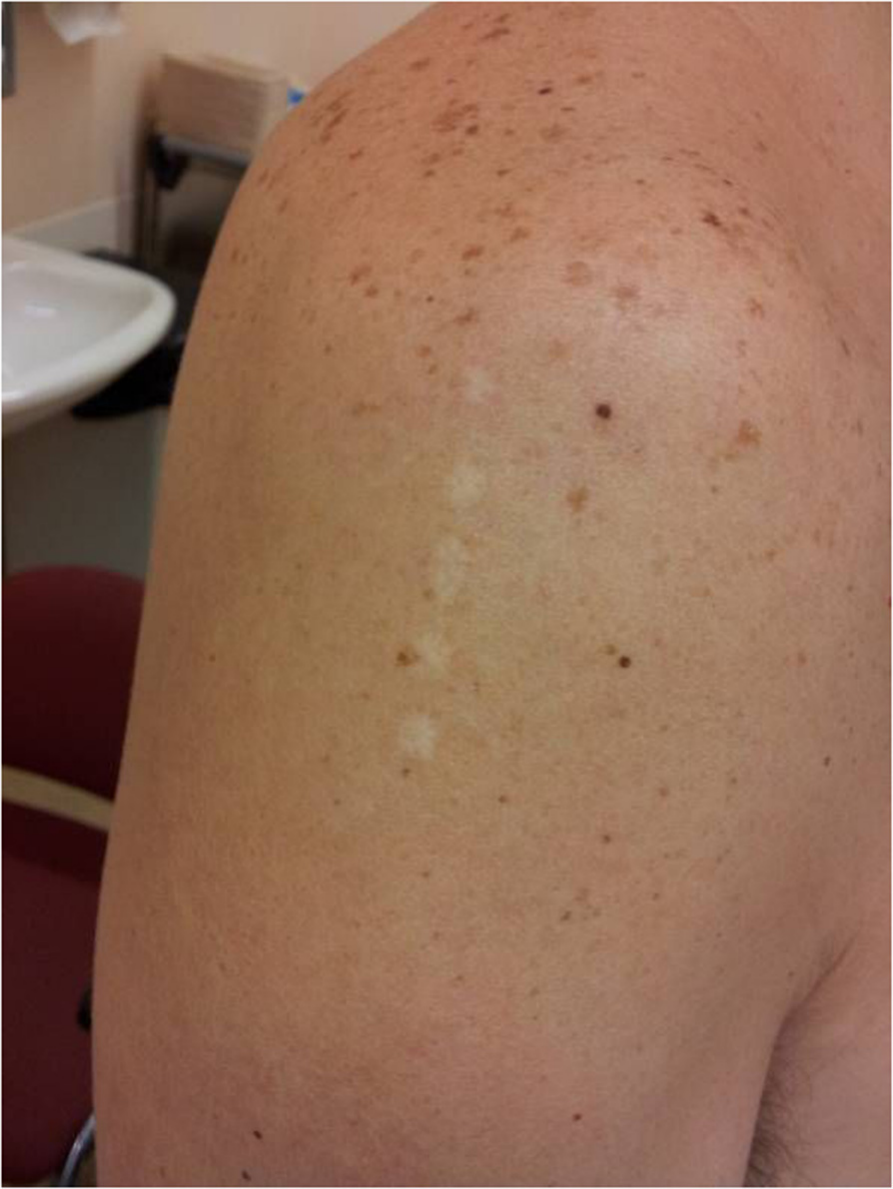
Scar consisting of regularly spaced dots on the right shoulder.

A 46-year-old man of Bolivian descent was seen at our surgical outpatient department for a lipoma on the right shoulder, which he wanted to remove. At physical examination, we saw not only a subcutaneous lipoma, but also a curious scar on the right shoulder (Figure 1), which consisted of five regularly spaced and aligned dots. Near this scar, remnants of a recent bruising could still be seen as a yellowish glow. Asked about the nature of the wound, our patient said that he had been in a so-called *Kambô* ritual a few months earlier at an indigenous tribe in the rainforests of northern Bolivia.

**Figure 2:**
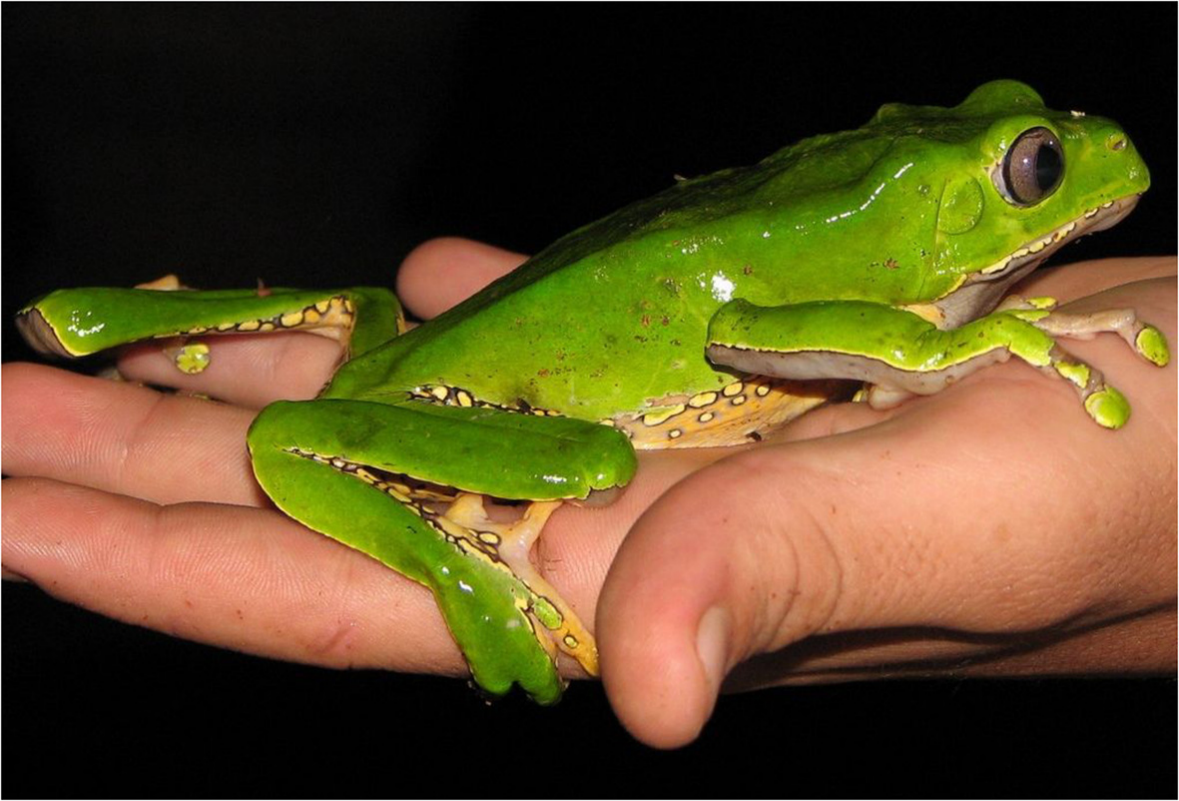
***Phyllomedusa bicolor*.**

Women are usually wounded on the leg or foot for the administration of *Phyllomedusa bicolor* secretion on the damaged skin (Figure 2). Within minutes heart throbbing, sweating and nausea arise that lead immediately to severe vomiting. After a few more minutes, the effects disappear. This purification ceremony, first described by Daly *et al.*[2], is not only used by local indigenous tribes, such as Matsés or Mayoruna, but also by urban people. It is said that the ritual brings luck to hunters, increases stamina and enhances physical and sexual strength [1,2]. The beneficial effects are not scientifically proven in randomized controlled trials, so the healing effect may be just a placebo effect.

The word *sapo* means frog in Portuguese and Spanish, whereas *kambô* is the common name of *Phyllomedusa bicolor* in South America. The frogs are found in the rainforests of Bolivia, Brazil, Colombia, Peru, French Guiana, Suriname and Venezuela near streams. To collect the secretion, the shaman carefully ties the frog by each leg into an X shape, next to an open fire. The secretion is carefully scraped off the skin, and left to dry on small sticks for storage. From these sticks, it will be applied to the burns. After this, the frog is released and returns to its natural habitat. The frog is never harmed, but always treated with utmost care and respect as the indigenous tribes believe that to harm it will anger the animal spirits.

All the peripheral and most of the central effects of the secretion – including tachycardia, dizziness, nausea, vomiting and sedation – are provoked by the high content of active peptides. These peptides include phyllocaerulein (hypotensive neuropeptide), phyllomedusin (a tachykinin which excite neurons, evokes behavioral responses, contracts smooth muscles and is a potent vasodilator and secretagogue), phyllokinin (induces relaxation of arterial smooth muscle by targeting bradykinin receptors), dermorphins (opiate-like activity) and deltorphins (opiate-like activity) [3].

The dried secretions of *Phyllomedusa bicolor* on wooden sticks are commercially available as “*Kambô* sticks” and are sold on markets and on the internet. Therefore, we want to inform doctors worldwide about the ritual of *Kambô* or *Sapo* as it is a type of voluntary envenomation. The ritual has no proven beneficial effects and no known influence on the long-term health of the person, but caution is required due to the toxicological aspects of the bioactive peptides and the possibility of infection of the wounds.

## Competing interests

The authors declare that there are no competing interests.
